# Application of group LASSO regression based Bayesian networks in risk factors exploration and disease prediction for acute kidney injury in hospitalized patients with hematologic malignancies

**DOI:** 10.1186/s12882-020-01786-w

**Published:** 2020-05-05

**Authors:** Yang Li, Xiaohong Chen, Yimei Wang, Jiachang Hu, Ziyan Shen, Xiaoqiang Ding

**Affiliations:** 1 Department of Nephrology, Zhongshan Hospital, Fudan University, No.180 Fenglin Road, Xuhui District, Shanghai, 200032 China; 2 Shanghai Medical Center of Kidney, Shanghai, 200032 China; 3 Shanghai Key Laboratory of Kidney and Blood Purification, Shanghai, 200032 China; 4 Shanghai Institute of Kidney and Dialysis, Shanghai, 200032 China; 5 Hemodialysis Quality Control Center of Shanghai, Shanghai, 200032 China

**Keywords:** Acute kidney injury, Hematologic malignancy, Bayesian networks, Disease prediction, Clinical epidemiology

## Abstract

**Background:**

Patients who were diagnosed with hematologic malignancies (HM) had a higher risk of acute kidney injury (AKI). This study applies the Bayesian networks (BNs) to investigate the interrelationships between AKI and its risk factors among HM patients, and to evaluate the predictive and inferential ability of BNs model in different clinical settings.

**Methods:**

During 2014 and 2015, a total of 2501 inpatients with HM were recruited in this retrospective study conducted in a tertiary hospital, Shanghai of China. Patients’ demographics, medical history, clinical and laboratory records on admission were extracted from the electronic medical records. Candidate predictors of AKI were screened in the group-LASSO (gLASSO) regression, and then they were incorporated into BNs analysis for further interrelationship modeling and disease prediction.

**Results:**

Of 2395 eligible patients with HM, 370 episodes were diagnosed with AKI (15.4%). Patients with multiple myeloma (24.1%) and leukemia (23.9%) had higher incidences of AKI, followed by lymphoma (13.4%). Screened by the gLASSO regression, variables as age, gender, diabetes, HM category, anti-tumor treatment, hemoglobin, serum creatinine (SCr), the estimated glomerular filtration rate (eGFR), serum uric acid, serum sodium and potassium level were found with significant associations with the occurrence of AKI. Through BNs analysis, age, hemoglobin, eGFR, serum sodium and potassium had directed connections with AKI. HM category and anti-tumor treatment were indirectly linked to AKI via hemoglobin and eGFR, and diabetes was connected with AKI by affecting eGFR level. BNs inferences concluded that when poor eGFR, anemia and hyponatremia occurred simultaneously, the patients’ probability of AKI was up to 78.5%. The area under the receiver operating characteristic curve (AUC) of BNs model was 0.835, higher than that in the logistic score model (0.763). It also showed a robust performance in 10-fold cross-validation (AUC: 0.812).

**Conclusion:**

Bayesian networks can provide a novel perspective to reveal the intrinsic connections between AKI and its risk factors in HM patients. The BNs predictive model could help us to calculate the probability of AKI at the individual level, and follow the tide of e-alert and big-data realize the early detection of AKI.

## Background

Patients with hematologic malignancies (HM) share a higher incidence of acute kidney injury (AKI) during anti-tumor treatment. A Danish population-based cohort study reported that the 1-year risk of AKI was 18.8% in patients diagnosed with lymphoma, 27.5% in leukemia and 31.8% in multiple myeloma [1]. Among these HM patients, the occurrence of AKI is not only associated with common risk factors in non-cancer patients but also with the malignancies itself and following treatment [2, 3]. The progression of AKI further limits anti-tumor treatment and brings about a higher in-hospital mortality and heavier economic burdens [4, 5]. Furthermore, AKI diagnosis is easily overlooked by physicians in other divisions apart from nephrology. A study in China found that about three-quarters of inpatients did not receive a prompt diagnosis of AKI during hospitalization [6].

Early recognition of high-risk patients with AKI could help us to adopt preventive strategies to reverse the development of AKI [7]. Several logistic regression-based models had been proposed to predict the occurrence of AKI in patients undergoing cardiac surgeries and other clinical settings [6, 8–11]. The precondition of logistic regression requires the variable independence. While risk factors of AKI are usually interdependent. Hence, developing a more flexible and efficient predictive model will facilitate the early recognition of AKI. Bayesian networks (BNs) is designed as a kind of machine-learning algorithm. It can not only display the complex networks among factors visually and graphically, but also acquire their probabilistic dependency relationships [12]. Moreover, BNs is not strict about statistical assumptions and perform well in handling the missing data. This made it more suitable for clinical researches [13]. Least absolute shrinkage and selection operator (LASSO) regression is an advanced variable selection algorithm for multi-collinear data or high-dimensional data. Previous studies proved that inserting LASSO regression into BNs analysis can not only simplify the complexity of the network but also improve the model’s predictive accuracy [14, 15].

In this study, we applied group LASSO regression-based Bayesian networks to investigate the interrelationships between AKI and its risk factors in HM patients, and to evaluate the predictive and inferential ability of BNs model in different clinical settings.

## Methods

### Study design and participants

During Oct. 1st, 2014 and Sept. 30th, 2015, a retrospective cohort study was conducted in Zhongshan Hospital of Fudan University, a tertiary hospital in eastern China. Patients who had a diagnosis of lymphoma, leukemia or multiple myeloma were enrolled as the study participants. Patients who hospitalized less than 24 h, underwent dialysis or renal replacement therapy (RRT) and lacked the repeated serum creatinine (SCr) tests were excluded from the final analysis [16, 17].

### Data collection

Patients’ demographic data, medical history, clinical diagnosis, anti-tumor treatment, biochemical tests, and other information were extracted from the hospital electronic medical records system and laboratory database. Baseline biochemical results refer to the first test within 24 h during hospitalization. We divided them into 3 parts: (1) Liver function: alanine aminotransferase (ALT), aspartate aminotransferase (AST) and total bilirubin (TBiL); (2) Renal function: SCr, the estimated glomerular filtration rate (eGFR) and serum uric acid (SUA); (3) Other: albumin, hemoglobin, white blood cell (WBC), serum sodium and potassium.

### Definition and classification

According to the KDIGO guideline in 2012 [18], AKI is defined as an absolute increase in SCr by ≥0.3 mg/dL within 48 h or ≥ 1.5-fold from the baseline within seven days. Since the urine output cannot be dated accurately, we only used the SCr changes for AKI diagnosis. The severity of AKI was divided into Stage 1: SCr increases ≥0.3 mg/dL or ≥ 1.5–fold to 1.9-fold baseline; Stage 2: SCr increases ≥2.9–3.0 fold baseline; Stage 3: SCr increases ≥3.0 fold baseline or ≥ 4.0 mg/dL, or the initiation of RRT [18]. According to the 10th revision of International Classification of Diseases (ICD-10), the hematologic malignancies in this study included lymphoma (C91-C95), leukemia (C81–85) and multiple myeloma (C90) [19]. Anti-tumor treatment was divided into autologous stem cell transplantation (ASCT), chemotherapy and untreated/palliative care. The baseline reference levels of serum sodium and potassium were 137~147 mmol/L and 3.5~5.3 mmol/L. Values below or above the reference level were defined as hypo−/hypernatremia and hypo−/hyperkalemia. The normal values of eGFR and SUA were set as ≥90 mL/min/1.73m^2^ and ≤ 359 μmol/L, respectively. Anemia refers to hemoglobin < 115 g/L, and hypoalbuminemia refers to albumin < 35 g/L.

### Group LASSO regression

The absolute shrinkage and selection operator (LASSO) is a shrinkage method within least square method that enables to shrink estimation of continuous variables towards zero [20]. In order to handle the categorical variable, the Group LASSO (gLASSO) is extensively developed to perform the predefined grouping variable selection instead of single dummy variable selection. Assuming that we have *J* groups of categorical variables {*G*_*1*_*,G*_*2*_*, …,G*_*j*_} and each of them had *p*_*1*_*,p*_*2*_*, … p*_*j*_ levels, the gLASSO estimator ${\hat{\beta}}^{GrLasso}$ is presented as:

$$ {\hat{\beta}}^{GrLasso}={\mathit{\arg}}_{\beta}\min \left\{\sum \limits_{i=1}^n\frac{1}{2}{\left({y}_i-\sum \limits_{j=1}^p{x}_{ij}{\beta}_j\right)}^2+ n\lambda \sum \limits_{j=1}^p\left\Vert {\beta}_j\right\Vert \right\} $$

By adjusting penalty *l*_*1*_ and *l*_*2*_, the candidate variables can be selected in group level and remain invariant in group orthogonal transformation such as ridge regression. The coefficients in one group will either all be zero or all nonzero. The penalty functions of *grLasso*, *grMCP*, and *grSCAD* carry out group selection, while the *gel* and *cMCP* penalties carry out bi-level selection. The point estimation of fitted lambda (λ) along with the regularization path is selected according to *AIC*, *BIC*, or *GCV* criteria. Then, k-fold cross-validation for penalized gLASSO models is performed to plot a grid of values for the regularization parameter lambda (λ). The *lambda.min* refers to the optimal variable selection with the minimum cross-validation error. Compared with the logistic model, gLASSO performs better on multi-collinear or high-dimensional data.

### Bayesian networks

The Bayesian networks (BNs) consists of two parts: a directed acyclic graph (DAG) and its subsequent conditional probability distribution (CPD). In the BNs, variables are graphically represented by the nodes *X = {X*_*i*_*, …, X*_*n*_*}* and the relationship between two nodes is connected by a unilateral arc. If the arc is going from *X*_*i*_ to *X*_*i + 1*_, we defined the *X*_*i*_ as the parent node and *X*_*i + 1*_ as the child node. CPD is acquired to quantify the probabilistic relationships between parent and child nodes. The global distribution factorization of *X* in BNs model could be specified as:

$$ P\left({X}_1,\dots, {X}_n\right)=P\left({X}_1\right)P\left({X}_2|{X}_1\right)\dots P\left({X}_n|{X}_1,{X}_2,\dots, {X}_{n-1}\right)=\prod \limits_1^nP\left({X}_i|\pi \left({X}_i\right)\right) $$

*π (X*_*i*_*)* refers to the set of the *X*_*i*_'s parent nodes *π (X*_*i*_*)∈{X*_*i*_*, …, X*_*n-1*_*}*, and the graphical separation refers to the conditional independence relationships between *(X*_*i*_*)* and *{X*_*i*_*, …, X*_*i-1*_*}*. BNs modeling contained structure learning and parameter learning. The structure learning is acquired from data and can be traced to 3 algorithms: constraint-based, score-based and hybrid algorithms. Parameter learning refers to applying either maximum likelihood (ML) estimation or Bayesian estimation method to compute the CPD of nodes in the established network. BNs inference is achieved by computing the posterior probability of *X* in the presence of new evidence *E*. When *E* changes, conditional probability distributions of both parent and child nodes are also affected. There are two algorithms for BNs inference, logical sampling algorithm and likelihood weighting algorithm, and the latter has a lower variance.

### Statistical analysis

Pearson chi-square test was used to compare the distribution differences of categorical variables and Cochran-Mantel-Haenszel (CMH) test was used for ordinal variables. The crude odds ratios (cOR) and its 95% confidence interval (CI) were calculated to quantify the association between factors and AKI. The analysis was run on IBM SPSS 22.0 (IBM Corp., Armonk, NY, USA), and the threshold of type I error (α) was set to 0.05. The process of variable selection in gLASSO regression was as follows: ① category variables were decomposed into dummy variables and their group label was assigned into another parallel dataset; ② the dummy and group datasets were analyzed in “grpreg” packages of R program 3.6.0 (R core team); ③ *grLasso* penalty and *BIC* criteria were used to estimate the fitted lambda (λ); ④ 10-fold cross-validation was performed to screen the optimal variable selection with the minimum cross-validation error. Then, the selected preditors further created a Bayesian network in “bnlearn” packages in the R program. The tabu-search algorithm was chosen to establish the BNs structure, and the ML method was used to acquire the CPD parameters. The area under the receiver operating characteristic curve (AUC) was applied to assess the prediction ability of the BNs model. A 10-fold cross-validation was also performed for internal validation and reducing the overfitting bias. The model diagram was drawn in Netica 5.18 (Norsys Software Corp., Vancouver, BC, Canada). Weka 3.8.0 (Waikato Environment for Knowledge Analysis, the University of Waikato, New Zealand) was used for model estimation.

## Results

During the study period, 2501 patients with hematologic malignancies were recruited. After excluding those unqualified participants, 2395 eligible patients were enrolled in the formal analysis (Supplement Figure 1). The average age of them was 54.9 ± 15.5 years old and 57.4% were male patients (*n* = 1375).

### AKI incidence and risk factors

A total of 370 (15.4%) episodes were diagnosed with AKI during hospitalization. Of them, 308(12.9%), 41(1.7%) and 21(0.9%) patients were located in AKI Stage 1, 2 and 3, respectively. Twenty patients require RRT. Stratified by HM category, the incidence of AKI in patients with multiple myeloma (24.1%) and leukemia (23.9%) was higher than that of lymphoma (13.4%).

As shown in Table 1, patients under 29 years old had the highest risk of AKI (cOR: 2.16). The AKI incidence was higher in female patients than in the male (18.2% vs. 13.4%). Pre-existing diabetes increased the likelihood of AKI, while such a correlation was not found in patients with hypertension. In comparison to untreated/palliative care, patients receiving ASCT and chemical treatment were more vulnerable to develop AKI (cOR: 4.37 and 2.24 respectively). Liver and renal dysfunction were also found to have a significant association with AKI. Patients with abnormal ALT, AST and SCr values on admission were more likely to develop AKI; insufficient eGFR and increased SUA level also increased the probability of AKI. Patients with initial anemia and hypoalbuminemia had a 2.72 fold and 3.85 fold increased risk of AKI.

**Table 1:** Associated factors of AKI in patients with hematologic malignancies

Variate	Total	AKI (%)	*χ*^*2*^	*p*-value	cOR (95%CI)
Age					
< 29 yr	213	57 (26.8)	0.564	0.453^a^	2.16 (1.47~3.18)
30~49 yr	539	78 (14.5)			1.00
50~69 yr	1236	172 (13.9)			0.96 (0.72~1.28)
≥ 70 yr	407	63 (15.5)			1.08 (0.76~1.55)
Gender					
Male	1375	184 (13.4)	10.561	0.001^b^	1.00
Female	1020	186 (18.2)			1.44 (1.16~1.80)
Comorbidities					
Hypertension	473	75 (15.9)	0.075	0.784^b^	1.04 (0.79~1.37)
Diabetes	814	159 (19.5)	15.748	< 0.001^b^	1.58 (1.26~1.98)
HM category					
Lymphoma	1941	261 (13.4)	31.429	< 0.001^b^	1.00
Leukemia	201	48 (23.9)			2.02 (1.42~2.86)
Multiple Myeloma	253	61 (24.1)			2.05 (1.49~2.81)
In-hospital Condition					
Emergent	163	41 (25.2)	12.610	< 0.001^b^	1.94 (1.34~2.82)
Normal	2232	329 (14.7)			1.00
Anti-tumor Treatment					
ASCT	50	13 (26.0)	20.846	< 0.001^b^	4.37 (2.04~9.36)
Chemotherapy	2036	334 (16.4)			2.24 (1.57~3.79)
Untreated/palliative	309	23 (7.4)			1.00
Liver Function					
ALT (≥40 U/L)	150	35 (23.3)	7.616	< 0.001^b^	1.74 (1.17~2.58)
AST (≥35 U/L)	270	62 (23.0)	13.154	< 0.001^b^	1.76 (1.29~2.39)
TBiL(≥20.4 μmol/L)	90	17 (18.9)	0.847	0.357^b^	1.29 (0.75~2.21)
Renal Function					
SCr(≥115 μmol/L)	113	79 (69.9)	269.309	< 0.001^b^	15.9 (10.44~24.21)
eGFR(≥90 mL/min/1.73m^2^)	1650	170 (10.3)	207.618	< 0.001^a^	1.00
eGFR(60~89 mL/min/1.73m^2^)	596	110 (18.4)			1.96 (1.51~2.55)
eGFR(≤59 mL/min/1.73m^2^)	147	90 (61.2)			13.75 (9.52~19.86)
SUA(≤359 μmol/L)	1619	198 (12.2)	88.388	< 0.001^a^	1.00
SUA (360~420 μmol/L)	398	58 (14.6)			1.22 (0.89~1.68)
SUA (421~480 μmol/L)	200	40 (20.0)			1.79 (1.23~2.62)
SUA (≥481 μmol/L)	178	74 (41.6)			5.11 (3.66~7.13)
Biochemical Test					
Album (< 35 g/L)	584	156 (26.7)	75.012	< 0.001^b^	2.72 (2.16~3.43)
Hemoglobin (< 115 g/L)	1338	297 (22.2)	105.701	< 0.001^b^	3.85 (2.93~5.04)
WBC (≥9.5 × 10^9^)	406	102 (25.1)	35.028	< 0.001^b^	2.15 (1.66~2.79)
Hyponatremia	325	135 (41.5)	216.335	< 0.001^b^	5.84 (4.50~7.59)
Hypernatremia	41	15 (36.6)			4.74 (2.47~9.09)
Hypokalemia	333	122 (36.6)	227.450	< 0.001^b^	4.59 (3.53~5.97)
Hyperkalemia	24	20 (83.3)			39.69 (13.45~117.15)

*AKI* acute kidney injury, *cOR* crude odds ratio, *HM* hematologic malignancy, *ASCT* autologous stem cell transplantation, *ALT* alanine aminotransferase, *AST* aspartate aminotransferase, *TBiL* total bilirubin, *SCr* serum creatinie, *eGFR* estimated glomerular filtration rate, *SUA* serum uric acid, *WBC* white blood cell

^a^ Cochran-Mantel-Haenszel (CMH) test; ^b^ Pearson Chi-square Test

### Variable selection in gLASSO

**Fig. 1:**
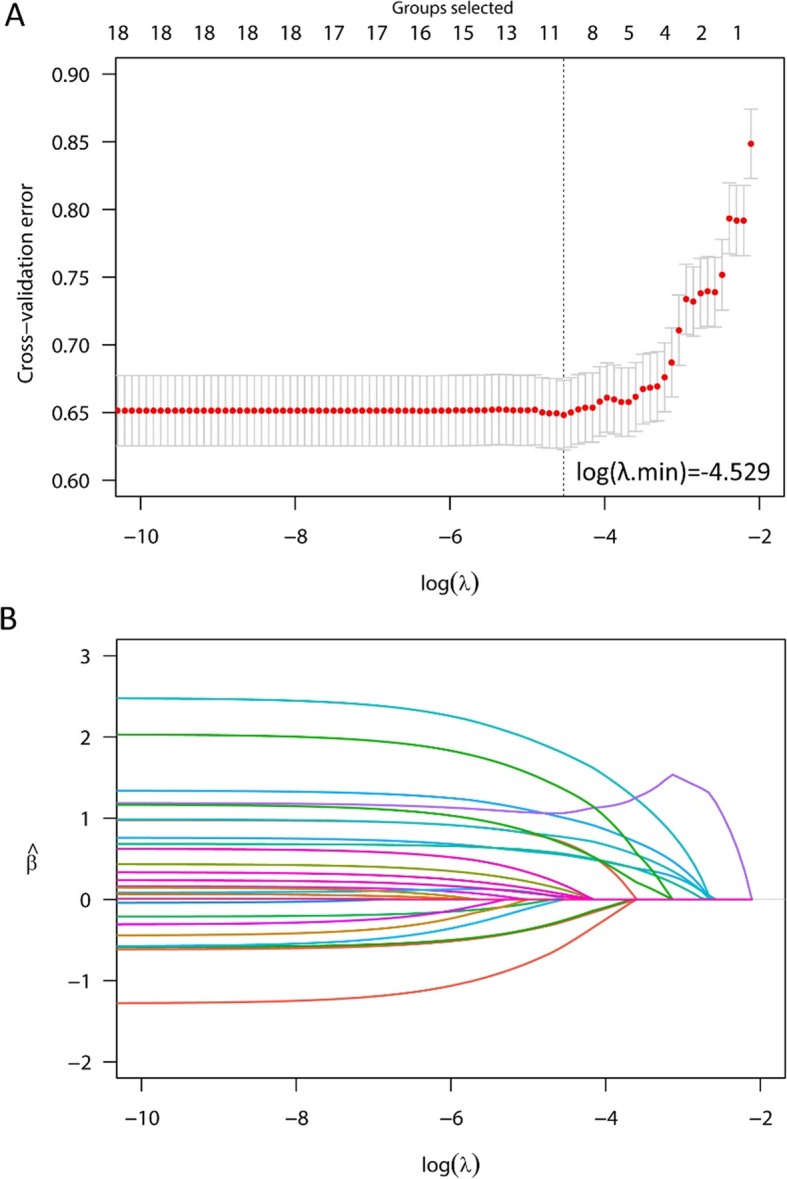
AKI variable selection by using gLASSO regression

The tuning parameter (λ) was specified in gLASSO regression by using 10-fold cross-validation in Fig. 1a. The optimal λ value was highlighted by the vertical lines with a minimizing cross-validation error. When log (λ) was equal to − 4.529, eleven of the initial nineteen variables were selected, including age, gender, diabetes, HM category, anti-tumor treatment, hemoglobin, SCr, eGFR, SUA, serum sodium and potassium levels. Figure 1b presented the gLASSO coefficient ($\hat{\beta}$) profiles of candidate variables. When the *gLASSO* model met *BIC* criteria(*λ* = 0.00896), the same predictors and their nonzero coefficients were identified.

### Bayesian network model of HM-related AKI

**Fig. 2:**
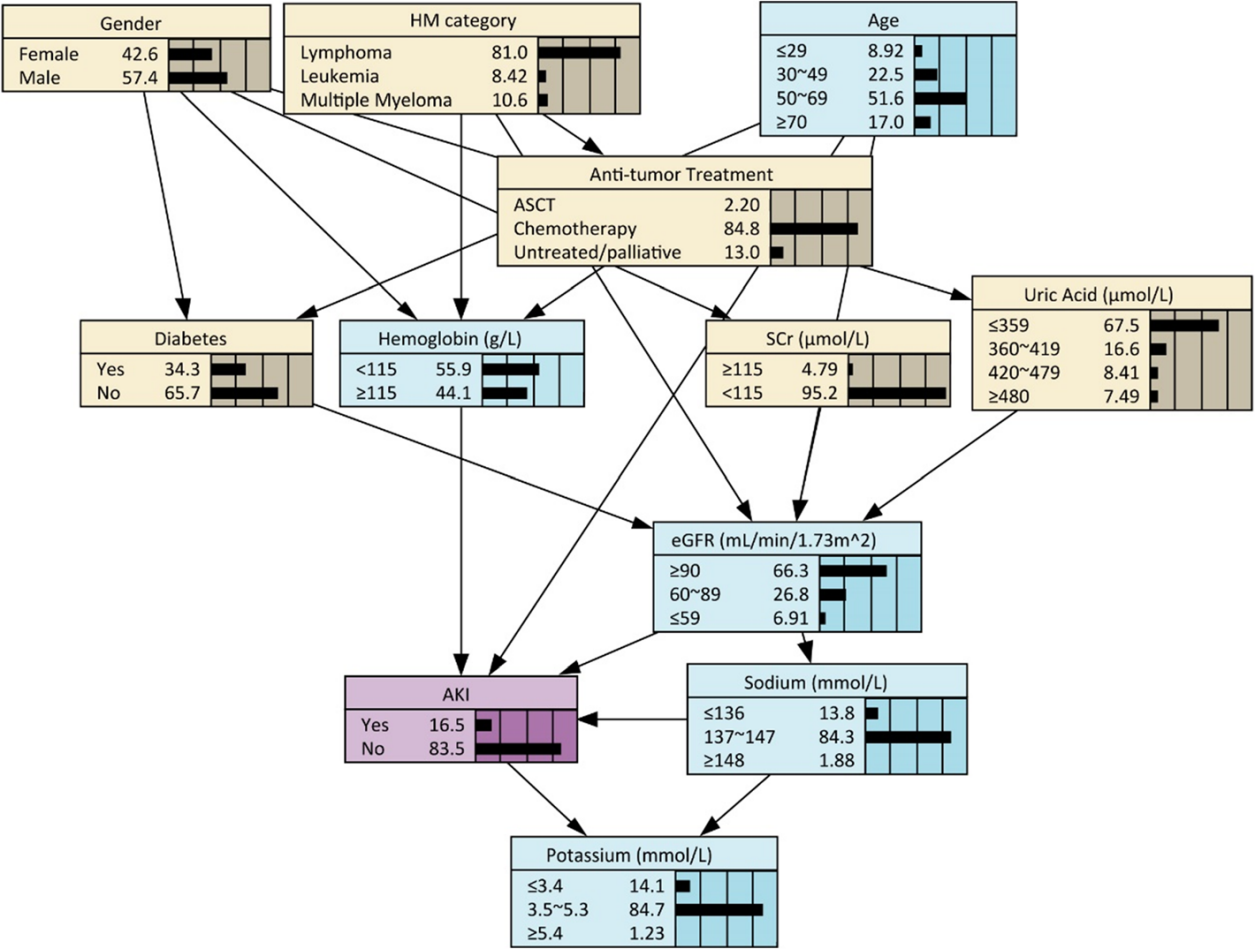
Bayesian Network model of factors relating to AKI in patients with HM

Though BNs analysis, we delineated the probabilistic dependencies between HM-related AKI and its preditors in a complex network (Fig. 2). It was observed that age, hemoglobin, eGFR, serum sodium and potassium created direct connections with AKI, while other variables were related to AKI indirectly. For instance, HM category and anti-tumor treatment indirectly linked with AKI via hemoglobin and eGFR, and diabetes had connected with AKI by affecting eGFR level. Moreover, the relationship between covariates can also be given in the network. Hemoglobin was related to gender, HM category and anti-tumor treatment; eGFR was influenced by age, diabetes, HM category, SCR and SUA level. Table 2 manifested the CPD table of AKI, quantifying the relationship between AKI and its parent nodes of eGFR, hemoglobin and serum sodium. Patients whose eGFR < 59 mL/min per 1.73 m^2^ together with anemia and hyponatremia shared the highest AKI incidence (78.5%). In a similar situation but hypernatremia, the probability of AKI was estimated to be 68.3%. In contrast, patients with normal eGFR, hemoglobin and sodium level had the lowest rate (5.2%).

**Table 2:** The conditional probability distribution of AKI with eGFR, hemoglobin and serum sodium as parent nodes

Parent Nodes			AKI incidence (%)	
eGFR	Hb	Sodium	Yes	No
≤59	<115	≤136	78.5%	21.5%
≤59	<115	137~147	47.6%	52.4%
≤59	<115	≥148	68.3%	31.7%
≤59	≥115	≤136	60.7%	39.3%
≤59	≥115	137~147	38.4%	61.6%
≤59	≥115	≥148	45.0%	55.0%
60~89	<115	≤136	44.7%	55.3%
60~89	<115	137~147	22.8%	77.2%
60~89	<115	≥148	42.8%	57.2%
60~89	≥115	≤136	19.2%	80.8%
60~89	≥115	137~147	12.0%	88.0%
60~89	≥115	≥148	33.4%	66.6%
≥90	<115	≤136	38.4%	61.6%
≥90	<115	137~147	10.3%	89.7%
≥90	<115	≥148	36.8%	63.2%
≥90	≥115	≤136	19.3%	80.7%
≥90	≥115	137~147	5.2%	94.8%
≥90	≥115	≥148	33.0%	67.0%

*AKI* acute kidney injury, *eGFR* estimated glomerular filtration rate, *Hb* hemoglobin

### Bayesian network evaluation and model inference

**Fig. 3:**
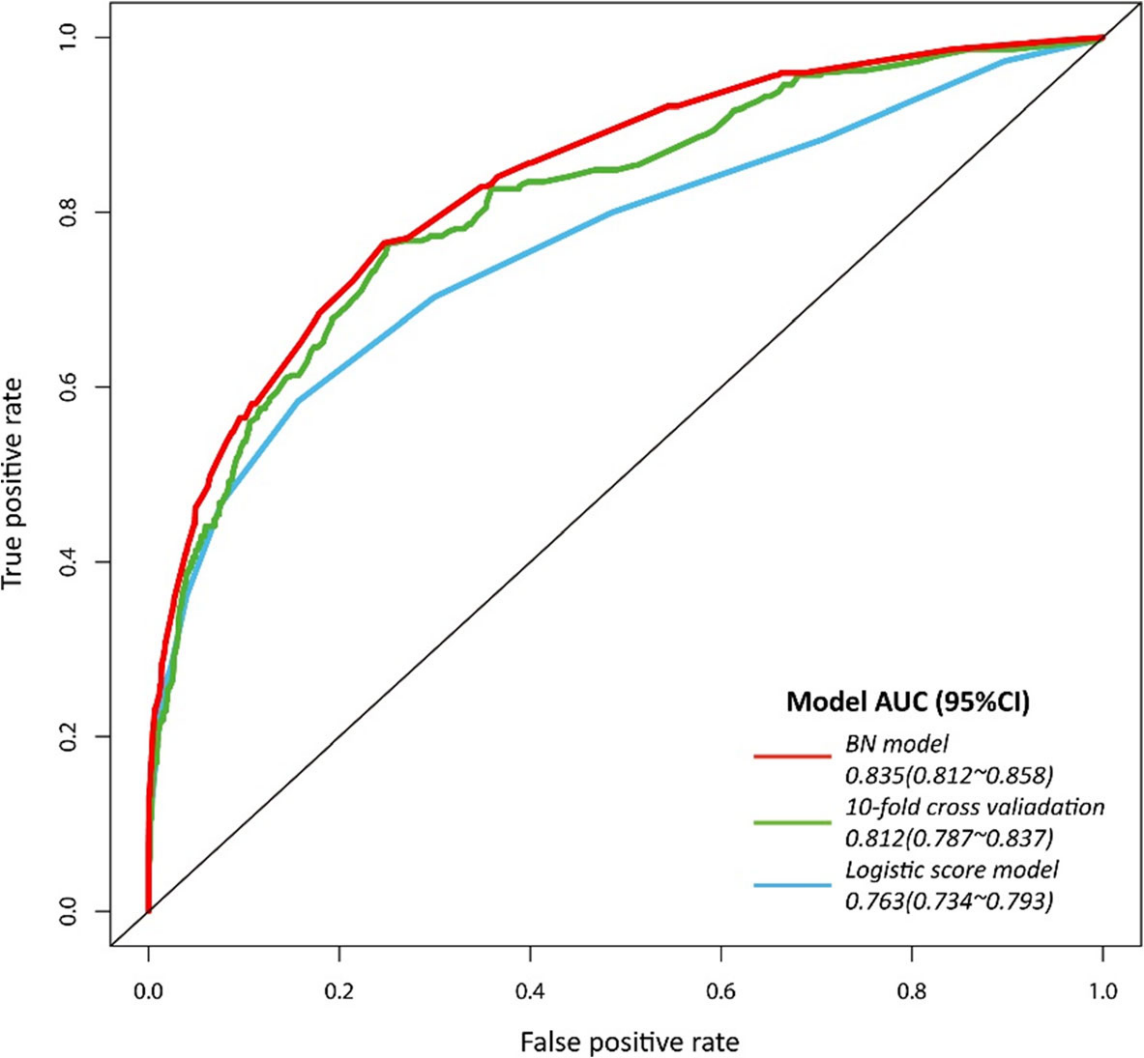
Receiver operating characteristic curves for AKI predictors in Bayesian network

**Fig. 4:**
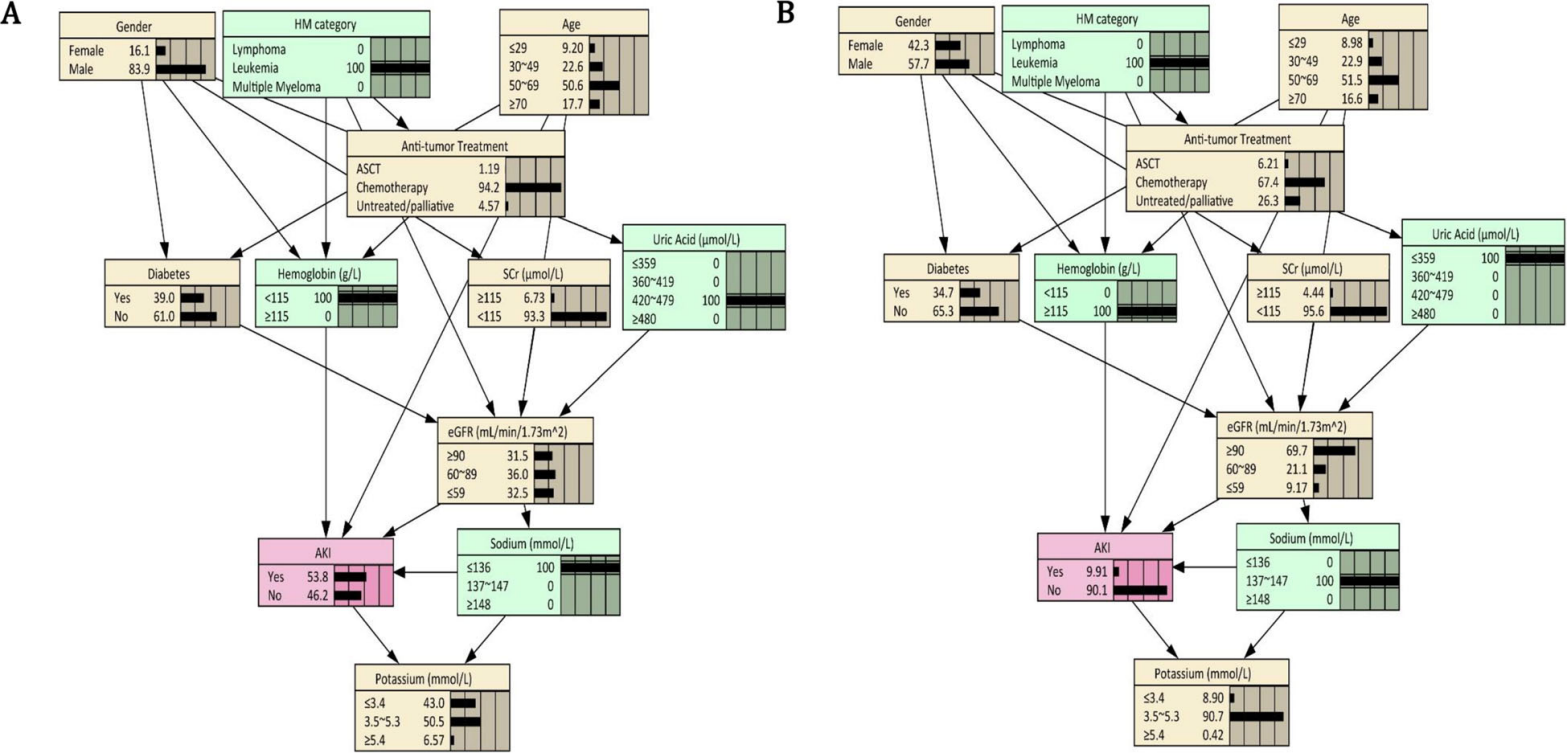
Bayesian network under known evidence variables

As shown in Fig. 3, the AUC value of BNs model was 0.835 (95% CI: 0.812 to 0.858), which was higher than that of the logistic score model (AUC = 0.763). In 10-fold cross-validation, the AUC maintained at the level of 0.812 (95% CI: 0.787 to 0.837). By using the Mantel-Haenszel test, no statistically significant difference in predictive accuracy was found between initial and cross-validation datasets (*p* = 0.298). According to the patients’ demographics and limited available clinical records, BNs could infer the individual probability of AKI occurrence during hospitalization. For instance, when anemia, hyperuricemia, and hyponatremia were initially found on admission in patients with leukemia, the expected probability of AKI was estimated to be 53.8% based on the prior information of BNs. However, once these biochemical indicators were corrected to the normal level in time, the risk of AKI can be reduced to 9.9% (Fig. 4).

## Discussion

With the development of novel chemotherapeutic agents and targeted medicine, the survival time and quality of life have been remarkably improved among cancer patients. Meanwhile, the periodic anti-tumor treatment also poses patients a higher risk of renal dysfunction [21]. In this study, the incidence of AKI among patients with multiple myeloma, leukemia and lymphoma was 24.1, 23.9 and 13.4%, respectively. It is higher than that of general inpatients [22–24] and patients with solid tumors [25, 26]. Therefore, it is essential to take measures to prevent AKI and adverse consequences associated with deterioration of renal function.

Developing the predictive models has been proved as a promising way for early detection of high-risk patients with AKI. While in the traditional logical regression, predictions can not be performed unless we know all the state of variables in the model. In fact, it is difficult to realize because persuading patients to accept excessive tests is against medical ethics. Thus, developing a more flexible model, which can handle the incomplete and missing data, may make more clinical senses. In this study, we applied the Bayesian network to AKI risk factor interpretation and risk prediction. It can also infer the probabilities of AKI with the finite amount of known evidence instead of the total. The parameters of unknown variables are computed by using the prior knowledge acquired from BNs modeling. It enables physicians to assess the patients’ individual AKI risk more flexibly and easily. We found that the AUC value of the BNs-based AKI model was higher than that of the logistic score model (0.835 vs. 0.763) and showed the strong robustness in 10-fold cross-validation. Moreover, the structure and parameters of BNs model are not fixed and can be optimized continuously by expanding the sample size and accumulating the variable information.

It was observed that the occurrence of HM-related AKI is usually multifactorial, including comorbidities, liver/renal dysfunction, anemia, HM category and anti-tumor treatment. The complex interrelationships between AKI and these risk factors make it unsuitable for the logistic analysis. Multicollinearity among variables is often encountered in clinical analysis and should be considered carefully unless it may lead to incorrect inferences. Penalization and regularization techniques, such as LASSO, have been proved to be the best algorithms for reducing the complexity of high-dimensional data. It is especially suitable for dealing with the enormous number of clinical factors and avoiding overfitting [27]. As an extension of LASSO method, gLASSO can implement grouping variable selection, which overcomes the limitations that LASSO can only select the single dummy variable. In the present study, we used gLASSO regression to screen 11 key predictors of AKI, and then present them for BNs structure and parameter learning. The pre-selection of variables before modeling can simplify the network structure and avoid the false positive arcs between two irrelevant nodes. Currently, LASSO, as an effective variable selection tool, has been widely used in machine learning modeling [28, 29].

Our results revealed that age, hemoglobin, eGFR, serum sodium and potassium were directly related to AKI. HM category and AKI was linked indirectly with hemoglobin and eGFR. Because of renal vascular dysfunction and chronic inflammation, patients with chronic kidney disease (CKD) are highly susceptible to AKI, which also can rapidly progress into a serious condition. Anemia is one of the most common complications in HM patients, which can be caused by the decreased hematopoietic capacity of bone marrow, blood dilution, repeated blood collection, iron metabolism dysfunction, decreased erythrocyte survival and a slow erythropoietin response et al. A Korean study reports that anemia was more common in HM patients than in patients with solid tumors (79.4% vs. 50.4%), and HM patients also share a higher risk of AKI and long-term mortality [30].

Apart from the conventional risk factors, our study reveals that electrolyte disturbance was also associated with a higher risk of AKI. *Olgar* et al. reported that among leukemia patients, hyponatremia and hypernatremia accounted for 11.7 and 9.5%, hypokalemia and hyperkalemia accounted for 7.6 and 6.0% [31]. Volume depletion such as hemorrhage, diarrhea and vomiting is the main cause of hyponatremia, which is not uncommon in HM patients receiving chemotherapy. Nutritional deficiency, and continuous undercapacity of volume can also result in hypokalemia. It was reported that the excessive production of blast cells can also cause hypokalemia in patients with leukemia [32]. Consistent with our study, the HM category is recognized to cast an effect on renal insufficiency [2]. Lymphomatous or leukemic infiltration can lead to enlarged kidneys. Leukemic hyperleukocytosis can alter the renal vascular permeability via microcapillary obstruction and renal vein thrombosis. in the presence of lymphadenopathy and drug-induced crystalluria, such as acyclovir and cotrimoxazole, obstructive nephropathy can occur. Moreover, we found that patients receiving ASCT had a higher risk of AKI. This may be related to the adverse effect of calcineurin inhibitors, graft versus host disease and hepatic sinusoidal obstruction syndrome [33].

If electrolytes monitor, risk factors recognition, and prophylaxis management were implemented properly, one in five hospitalized AKI can be avoided [34]. The BNs model established in this study can be used to infer the probability of AKI, so as to identify high-risk patients in advance and guide subsequent preventive treatment. When leukemia patients were initially diagnosed with anemia, hyperuricemia, and hyponatremia, the expected probability of AKI was 53.8%. If these biochemical indicators were corrected to normal level timely, the incidence of AKI would be significantly reduced to 9.9%.

Our study is the first application of BNs in the AKI study field. It provides us a novel perspective to interpret the interactions between AKI and its risk factors. BNs model also shows a superior predictive ability, which can realize accurate probability calculation at individual levels. Nevertheless, the study’s limitations should be illustrated. Firstly, the participants of this study came from a single medical center, which may affect the sample representation. Secondly, the lack of data on nephrotoxic drugs may underestimate the association between chemical treatment and AKI. Thirdly, data in this study was extracted from the medical record system. Arcs in BNs can only represent the probability dependencies, and the causal reasoning needs to be further verified in a prospective cohort in combination with professional knowledge.

## Conclusions

AKI is prevalent in hospitalized patients with HM, influenced by a variety of factors including comorbidity, renal/liver dysfunction and anti-tumor treatment. Bayesian networks can reveal the inherent connections between HM-related AKI and its multiple risk factors. The BNs predictive model could help us to calculate the probability of AKI at the individual level, and follow the tide of e-alert and big-data realize the early detection of AKI.

## Supplementary information

**Additional file 1: Supplement Figure S1.** Flow chart of the study population selection.

## Data Availability

Data can be available by contacting the corresponding author.

## Abbreviations

AKI: Acute kidney injury
ALT: Alanine aminotransferase;
ASCT: Autologous stem cell transplantation
AST: Aspartate aminotransferase
AUC: The area under the receiver operating characteristic curve
BNs: Bayesian networks
CI: Confidence interval
CKD: Chronic kidney disease
CPD: Conditional probability distribution
cOR: Crude Odds ratios
eGFR: Estimated Glomerular filtration rate
gLASSO: Group Least absolute shrinkage and selection operator
HM: Hematologic malignancies
LASSO: Least absolute shrinkage and selection operator
ML: Maximum likelihood
RRT: Renal replacement therapy
SCr: Serum creatinine
SUA: Serum uric acid
TBiL: Total bilirubin
WBC: White blood cell

## References

[1] Christiansen CF, Johansen MB, Langeberg WJ, Fryzek JP, Sørensen HT (2011). Incidence of acute kidney injury in cancer patients: a Danish population-based cohort study. Eur J Intern Med.

[2] Canet E, Vincent F, Darmon M, Soares M (2015). Acute kidney injury in hematological patients. Curr Opin Crit Care.

[3] Harris KP, Hattersley JM, Feehally J, Walls J (1991). Acute renal failure associated with haematological malignancies: a review of 10 years experience. Eur J Haematol.

[4] Lahoti A, Nates JL, Wakefield CD, Price KJ, Salahudeen AK (2011). Costs and Outcomes of acute kidney injury in critically ill patients with Cancer. J Support Oncol.

[5] Canet E, Zafrani L, Lambert J, Thieblemont C, Galicier L, Schnell D, Raffoux E, Lengline E, Chevret S, Darmon M (2013). Acute kidney injury in patients with newly diagnosed high-grade hematological malignancies: impact on remission and survival. PLoS One.

[6] Yang L, Xing G, Wang L, Wu Y, Li S, Xu G, He Q, Chen J, Chen M, Liu X. Acute kidney injury in China: a cross-sectional survey. Lancet (London, England). 2015;386(10002):1465–71. 10.1016/S0140-6736(15)00344-X 26466051

[7] Wang Y, Fang Y, Teng J, Ding X (2016). Acute kidney injury epidemiology: from recognition to intervention. Contrib Nephrol.

[8] Palomba H, de Castro I, Neto AL, Lage S, Yu L (2007). Acute kidney injury prediction following elective cardiac surgery: AKICS score. Kidney Int.

[9] Jiang W, Teng J, Xu J, Shen B, Wang Y, Fang Y, Zou Z, Jin J, Zhuang Y, Liu L et al. Dynamic Predictive Scores for Cardiac Surgery-Associated Acute Kidney Injury. J Am Heart Assoc. 2016;5(8):e003754. 10.1161/JAHA.116.003754 PMC5015294 27491837

[10] Kim WH, Lee SM, Choi JW, Kim EH, Lee JH, Jung JW, Ahn JH, Sung KI, Kim CS, Cho HS (2013). Simplified clinical risk score to predict acute kidney injury after aortic surgery. J Cardiothorac Vasc Anesthesia.

[11] Madhavan MV, Généreux P, Rubin J, Palmerini T, Caixeta A, Xu K, Weisz G, Mehran R, Stone GW (2014). Usefulness of the SYNTAX score to predict acute kidney injury after percutaneous coronary intervention (from the acute catheterization and urgent intervention triage strategy trial). Am J Cardiol.

[12] Scutari M (2010). Learning Bayesian networks with the bnlearn R package. J Stat Softw.

[13] Fuster-Parra P, Tauler P, Bennasar-Veny M, Ligeza A, Lopez-Gonzalez AA, Aguilo A (2016). Bayesian network modeling: a case study of an epidemiologic system analysis of cardiovascular risk. Comput Methods Prog Biomed.

[14] McNally RJ, Mair P, Mugno BL, Riemann BC (2017). Co-morbid obsessive-compulsive disorder and depression: a Bayesian network approach. Psychol Med.

[15] Zhang T, Ma Y, Xiao X, Lin Y, Zhang X, Yin F, Li X (2019). Dynamic Bayesian network in infectious diseases surveillance: a simulation study. Sci Rep.

[16] Levey AS, de Jong PE, Coresh J, El NM, Astor BC, Matsushita K, Gansevoort RT, Kasiske BL, Eckardt KU (2011). The definition, classification, and prognosis of chronic kidney disease: a KDIGO controversies conference report. Kidney Int.

[17] Xu X, Nie S, Liu Z, Chen C, Xu G, Zha Y, Qian J, Liu B, Han S, Xu A (2015). Epidemiology and clinical correlates of AKI in Chinese hospitalized adults. Clin J Am Soc Nephrol Cjasn.

[18] Disease K, Outcomes IG (2012). Acute kidney injury work group: KDIGO clinical practice guideline for acute kidney injury. Kidney Int Suppl.

[19] International Statistical Classification of Diseases and Related Health Problems 10th Revision, https://icd.who.int/browse10/2016/en. Accessed 5 Feb 2017.

[20] Tibshirani R (1996). Regression shrinkage and selection via the Lasso. J R Stat Soc.

[21] Susantitaphong P, Cruz DN, Cerda J, Abulfaraj M, Alqahtani F, Koulouridis I, Jaber BL (2013). World incidence of AKI: a meta-analysis. Clin J Am Soc Nephrol Cjasn.

[22] Fang Y, Ding X, Zhong Y, Zou J, Teng J, Tang Y, Lin J, Lin P (2010). Acute kidney injury in a Chinese hospitalized population. Blood Purif.

[23] Cheng X, Wu B, Liu Y, Mao H, Xing C (2017). Incidence and diagnosis of acute kidney injury in hospitalized adult patients: a retrospective observational study in a tertiary teaching Hospital in Southeast China. BMC Nephrol.

[24] Pavkov ME, Harding JL, Burrows NR (2018). Trends in hospitalizations for acute kidney injury - United States, 2000-2014. MMWR Morb Mortal Wkly Rep.

[25] Riffaut N, Moranne O, Hertig A, Hannedouche T, Couchoud C. Outcomes of acute kidney injury depend on initial clinical features: a national French cohort study. Nephrol Dial Transplant. 2018;33(12):2218-27. 10.1093/ndt/gfy137 29846676

[26] Salahudeen AK, Doshi SM, Pawar T, Nowshad G, Lahoti A, Shah P (2013). Incidence rate, clinical correlates, and Outcomes of AKI in patients admitted to a Comprehensive Cancer Center. Clin J Am Soc Nephrol Cjasn.

[27] Hepp T, Schmid M, Gefeller O, Waldmann E, Mayr A (2016). Approaches to regularized regression - a comparison between gradient boosting and the Lasso. Methods Inf Med.

[28] Goto T, Camargo CA, Faridi MK, Yun BJ, Hasegawa K (2018). Machine learning approaches for predicting disposition of asthma and COPD exacerbations in the ED. Am J Emerg Med.

[29] Xu H, Zhao X, Shi Y, Li X, Qian Y, Zou J, Yi H, Huang H, Guan J, Yin S (2019). Development and validation of a simple-to-use clinical nomogram for predicting obstructive sleep apnea. BMC Pulm Med.

[30] Han SS, Baek SH, Ahn SY, Chin HJ, Na KY, Chae DW, Kim S (2015). Anemia is a risk factor for acute kidney injury and long-term mortality in critically ill patients. Tohoku J Exp Med.

[31] Olgar S, Yetgin S, Cetin M, Aras T, Akhan O (2005). Electrolyte abnormalities at diagnosis of acute lymphocytic leukemia may be a clue for renal damage in long-term period. J Pediatr Hematol Oncol.

[32] Adams PC, Woodhouse KW, Adela M, Parnham A (1981). Exaggerated hypokalaemia in acute myeloid leukaemia. Br Med J (Clin Res Ed).

[33] Andronesi AG, Tanase AD, Sorohan BM, Craciun OG, Stefan L, Varady Z, Lipan L, Obrisca B, Truica A, Ismail G (2019). Incidence and risk factors for acute kidney injury following autologous stem cell transplantation for multiple myeloma. Cancer medicine.

[34] Mayor S (2009). UK report into acute kidney injury deaths urges electrolyte checks in all emergency admissions. BMJ.

