# Supplementary figures and images for: Application of group LASSO regression based Bayesian networks in risk factors exploration and disease prediction for acute kidney injury in hospitalized patients with hematologic malignancies
Source: BMC Nephrol. 2020 May 5;21:162. doi: 10.1186/s12882-020-01786-w (PMC7201633; doi:10.1186/s12882-020-01786-w)

**Supplement Figure 1.** Flow chart of the study population selection**
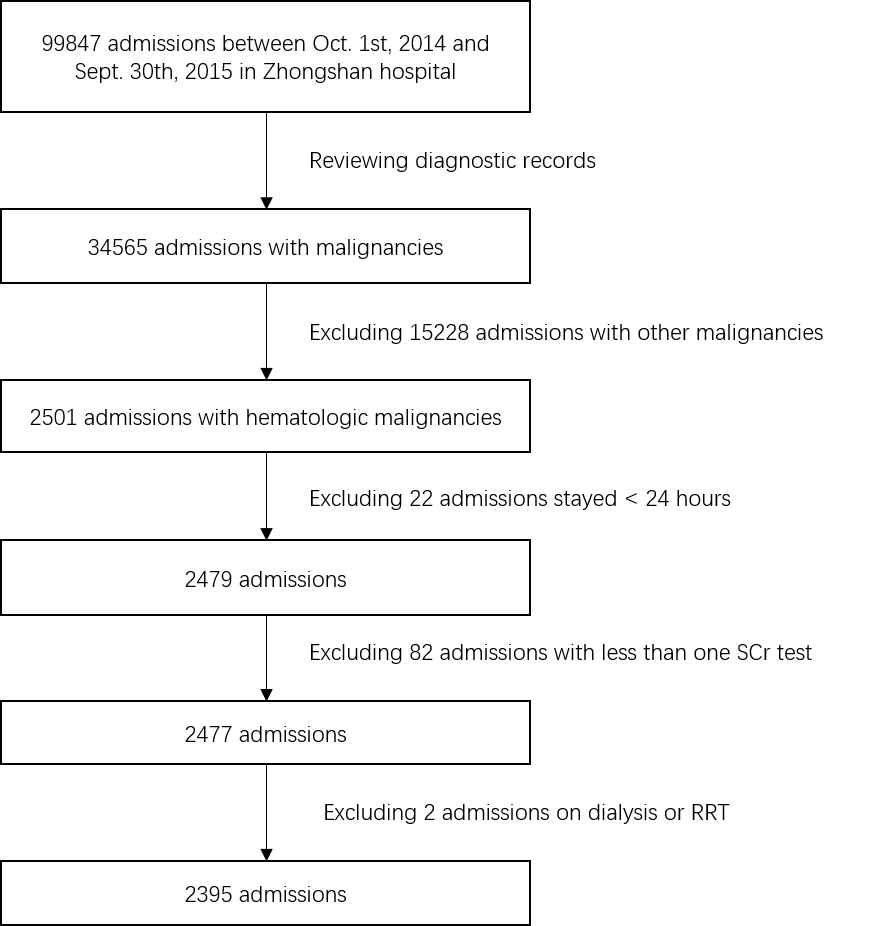
**

Supplement: Supplementary file 1 — Additional file 1: Supplement Figure S1. Flow chart of the study population selection. [file 12882_2020_1786_MOESM1_ESM.docx]
